# Efficacy of Anti-Leishmania Therapy in Visceral Leishmaniasis among HIV Infected Patients: A Systematic Review with Indirect Comparison

**DOI:** 10.1371/journal.pntd.0002195

**Published:** 2013-05-02

**Authors:** Gláucia F. Cota, Marcos R. de Sousa, Tatiani Oliveira Fereguetti, Ana Rabello

**Affiliations:** 1 Laboratory of Clinical Research, Centro de Pesquisas René Rachou, Fundação Oswaldo Cruz, Fiocruz, Belo Horizonte, Minas Gerais, Brazil; 2 Eduardo de Menezes Hospital, Fundação Hospitalar do Estado de Minas Gerais-FHEMIG, Belo Horizonte, Minas Gerais, Brazil; 3 Post-Graduate Program in Adult Health Sciences, Federal University of Minas Gerais, Belo Horizonte, Minas Gerais, Brazil; RTI International, United States of America

## Abstract

**Objective:**

We conducted a systematic literature review with indirect comparison of studies evaluating therapeutic efficacy and toxicity associated to visceral leishmaniasis (VL) therapy among HIV infected individuals.

**Main outcome measurements:**

The outcomes of interest were clinical and parasitological cure, mortality, and adverse events.

**Methods:**

PRISMA guidelines for systematic reviews and Cochrane manual were followed. Sources were MEDLINE, LILACS, EMBASE, Web of Knowledge databases and manual search of references from evaluated studies. We included all studies reporting outcomes after VL treatment, regardless of their design. Study quality was evaluated systematically by using the Newcastle-Ottawa Scale (NOS) for assessing the quality of nonrandomized studies in meta-analyses. Comprehensive Meta-Analysis software v.2.2.048 was used to perform one-group meta-analysis of study arms with the same drug to estimate global rates of success and adverse events with each drug. These estimates were used, when possible, to indirectly compare treatment options, adjusted for CD4 count. Direct comparison was pooled when available.

**Results:**

Seventeen studies reporting five treatment regimens and outcome of 920 VL episodes occurring in HIV infected individuals were included. The main outstanding difference in outcome among the treatment regimens was observed in mortality rate: it was around 3 times higher with high-dose antimony use (18.4%, CI 95% 13.3–25%), indirectly compared to lipid formulations of amphotericin B treatment (6.1%, CI 95% 3.9–9.4%). It was observed, also by indirect comparison, higher rates of clinical improvement in study arms using amphotericin B than in study arms using pentavalent antimonial therapy (Sb^v^). The parasitological cure, an outcome that presented some degree of risk of selection and verification bias, had rates that varied widely within the same treatment arm, with high heterogeneity, hampering any formal comparison among drugs. One direct comparison of amphotericin and antimoniate was possible combining results of two studies and confirming the superiority of amphotericin.

**Conclusions:**

Available evidence suggests that amphotericin is superior to antimony treatment. Death rate using antimoniate high dose is unacceptably high. Randomized controlled trials are necessary to compare different formulations and doses of amphotericin, alternative therapies and drug combinations.

## Author Summary

In co-infection with HIV/AIDS, visceral leishmaniasis (VL) most often results in an unfavorable response to treatment, frequent relapses, and in premature deaths. Scarce data is available regarding the treatment of leishmaniasis in HIV-infected patients (VL-HIV). Despite this, clinical decisions must be made. To aid in this task we reviewed comprehensive and systematically the available literature about efficacy and toxicity of therapeutic options for VL-HIV. PRISMA guidelines and Cochrane manual for systematic reviews were followed. Direct and indirect comparisons of nonrandomized studies were used, adjusting for CD4 count. Seventeen studies reporting five treatment regimens and outcome of 920 VL episodes occurring in HIV infected individuals were included. Results suggest higher survival and clinical response rate with amphotericin B than with antimony treatment. Antimonial therapy carries a higher rate of drug discontinuation and a significantly higher mortality indirectly compared to treatment with amphotericin B. Randomized controlled trials are needed to compare doses and formulations of amphotericin and alternative treatments.

## Introduction

In recent years, several reports have emphasized the increasing importance of visceral leishmaniasis (VL) as an opportunistic infection among HIV-positive patients in areas where both infections are endemic [1]. Chemotherapeutic agents with efficacy against VL include amphotericin B, pentavalent antimonial drugs, paramomycin (a parenteral aminoglycoside) and miltefosine (the first oral drug for treatment of VL). The pentavalent antimonial drugs (Sb^v^), sodium stibogluconate (SSG) and meglumine antimoniate have been used for past decades as the first line drugs for treatment because of their low cost and availability in most countries. Currently this option has been sidelined since more efficacious and less toxic alternatives exist [2]. Amphotericin B deoxycholate has high antileishmanial efficacy but it is associated with high risk of renal toxicity and other side effects and has been replaced in recent years in countries with sufficient financial resources by lipidic formulations of the drug. Pentamidine isethionate, a second-line alternative treatment is rarely used due to suboptimal efficacy and toxicity [3]. Although VL is treated similarly in patients with and without HIV infection, co-infected patients generally have low cure and high mortality rates [4], [5]. Furthermore, HIV-infected patients are more likely to suffer treatment-related adverse events than the HIV-negative population [6], [7].

Despite the prevalence, clinical implications and epidemiological impact of *Leishmania*/HIV co-infection, surprisingly scarce data is available regarding the treatment of leishmaniasis in HIV-infected patients. Major challenges include widespread resistance to pentavalent antimonial compounds, high treatment failure, toxicity and relapse rates [8]. The optimal therapy, including duration and dosages remain to be established. Indirect comparisons of nonrandomized studies are useful in the absence of randomized controlled trials, knowing its limitations and its assumptions [9], [10], adjusting for important covariates such as CD4 lymphocyte count. The aim of this study is to perform a systematic literature review regarding therapeutic efficacy and toxicity associated with visceral leishmaniasis therapy among HIV infected individuals, making comparisons of treatment options when possible.

## Methods

The review methodology followed the recommendations published by PRISMA guidelines [11] for systematic reviews and Cochrane Collaboration Group [12]. The search was done until 30, September 2012. A literature search was performed in PubMed, EMBASE, LILACS and WEB OF KNOWLEDGE databases using the search terms ‘visceral leishmaniasis’, ‘HIV infections’, ‘therapy’, without language or publication status restrictions.

Studies were included if reporting response to therapy to VL occurring in individuals with HIV infection. Literature search was not limited to randomized controlled trials, including all study designs. Studies involving less than ten patients; or containing a mixed population where data from HIV-infected patients or from different treatment arms could not be extracted separately were excluded. Study selection was made independently by two reviewers (GFC, TOF) and any disagreement was resolved by consensus or by discussion with a third reviewer (AR).

The outcomes of interest were clinical and parasitological cure, global response [defined as initial parasitological clearance in combination with clinical improvement, or clinical cure alone for patients for whom a test of cure (TOC) could not be performed], early mortality (reported death during or until 30 days after treatment), treatment interruption due to intolerance and adverse event rate and relapse rate.

The selected articles were read in full to confirm eligibility and to extract data. Data extraction and quality assessment were carried out by one author and checked by a second reviewer (TOF). The following information was recorded: country; year of publication and design of the study, patient characteristics (age, gender, CD4 lymphocyte count, antiretroviral use), rate of primary VL, treatment schedule, VL diagnostic criteria, VL cure criteria (clinical and/or parasitological), loss of following, clinical and parasitological cure rate. The follow-up length and relapse rate were also recorded.

The Newcastle-Ottawa Scale (NOS) [13] was used to assess the quality of nonrandomized studies. Using this ‘star system’ each included study was judged on three broad perspectives: the selection of the study groups; the comparability of the groups; and the ascertainment of outcome of interest.

### Statistical analysis

Comprehensive Meta-Analysis software v.2.2.048 was used to perform one-group meta-analysis of study arms with the same drug to estimate pooled rates of success and adverse events with each drug. These estimates were used, when possible, to indirectly compare treatment options. These unadjusted indirect comparisons were confronted with direct comparisons, when available. Also, the indirect comparisons were adjusted for CD4 lymphocyte count by using meta-regression. Meta-regression was used to explore the relationship between event rate and CD4 count by using mixed effects regression (unrestricted maximum likelihood). We used the Inconsistency (*I^2^*) statistic to evaluate heterogeneity. If significant heterogeneity was found, the results from the random effects model were emphasized and summary measures were analyzed as limited information, looking for differences in studies. Random effects model is a strategy that allows the heterogeneity inter-study would be incorporated through a broad confidence interval, generating a more conservative estimate of the measure of the effect. Clinical cure, global response and death rates analysis were performed according to the intention-to-treat analysis: the analysis was based on the total number of randomly assigned participants, irrespective of how the original study investigators analyzed the data. Publication bias was assessed by Egger's test [14].

## Results

**Figure 1:**
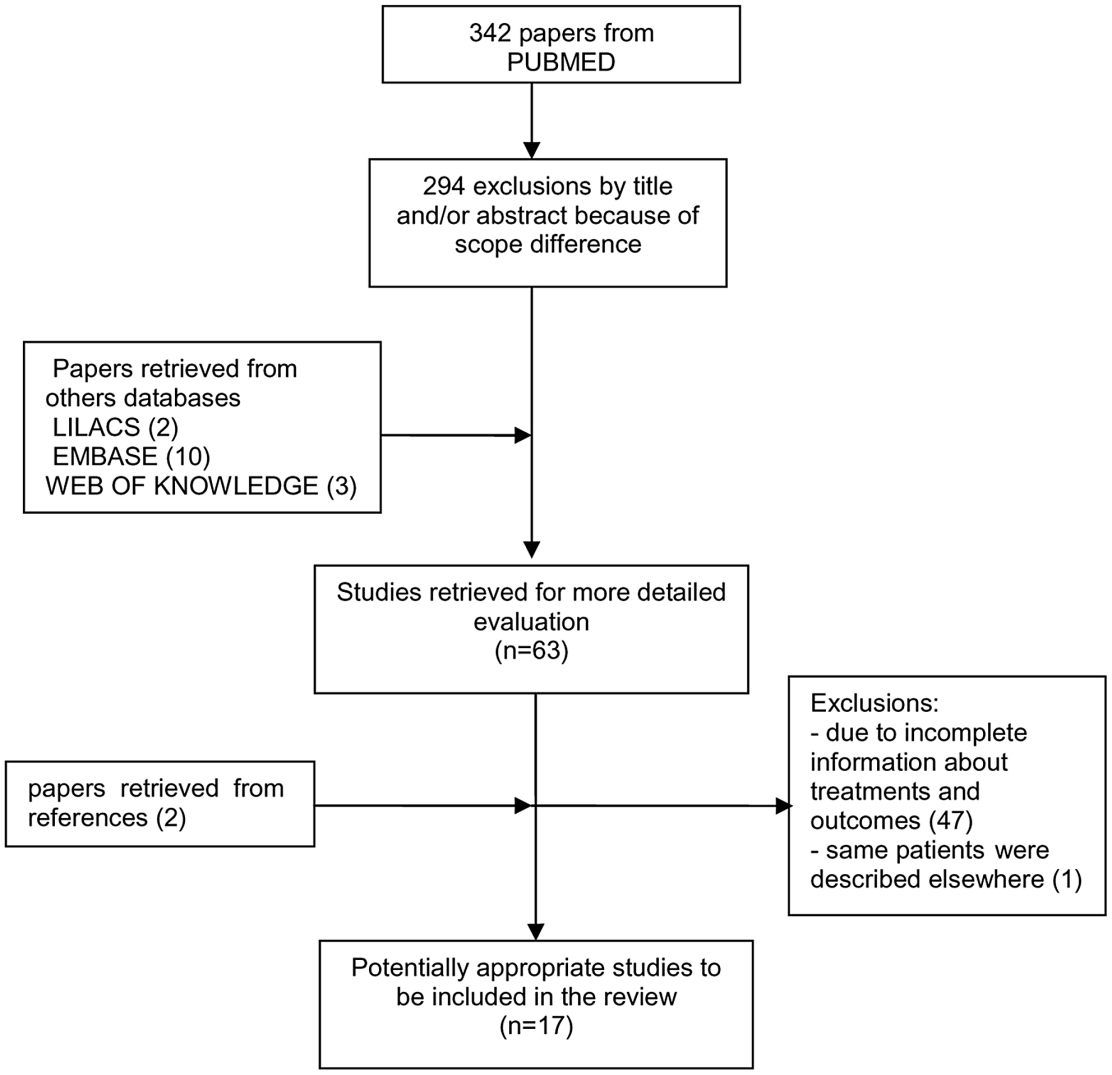
Study selection process.

Our search identified 342 articles from PubMed. EMBASE, LILACS and WEB OF KNOWLEDGE databases added 10, 2 and 3 papers respectively. After exclusions by title and abstracts, 63 potentially relevant papers were selected for full text evaluation (Figure 1). Two other titles were identified from references of the primary manuscripts. Of these 65 studies, 47 papers were excluded due to incomplete information about treatment and/or outcomes, including two congresses abstracts. One paper [15] was excluded because same patients were described elsewhere [16]. In a study [17], one treatment arm was also excluded by overlapping publication [18]. Thus, we included 17 articles [6], [16]–[31] involving 920 VL episodes among HIV-infected patients. Although in 4 studies the percentage of VL primary and relapse was not informed, most included patients had first VL episodes (76.1%). From 17 studies, only four were randomized trials [6], [22], [23], [27] involving 279 participants. However, these trials compared different treatment arms. We found only two studies [29], [30], nonrandomized, compared the same treatment regimens (amphotericin *versus* low dose antimony) and thus allow direct comparison. The methodological characteristics of studies, namely inclusion and exclusion, VL disease, clinical improvement and TOC criteria are shown in Tables 1 and 2. Three papers were prospective non comparative studies and other nine were historic cohort studies with one or more treatment regimen arms reported.

**Table 1:** Studies characteristics: design, treatment schedules and therapy arms with available data.

Author, year, country	Treatment arms (number of treated episodes)	Treatment regimens	Study design
Ritmeijer, 2011, Ethiopia	Liposomal amphotericin B (195)	total dose of 30 mg/kg, intravenously, divided into 6 infusions of 5 mg/kg on alternate days	Retrospective, historic cohort non comparative, multicenter
Sinha, 2011, India	Liposomal amphotericin B (55)	total dose of 20 mg/kg, intravenously, divided into 4 infusions of days 1, 2, 5, and 10 (or patients relapsing after having previously received a full course of liposomal amphotericin B, a total dose of 25 mg/kg was given in 5 doses: days 1, 2, 5, 10, and 15)	Retrospective, historic cohort non comparative, multicenter
Molina, 2007, Spain	Liposomal amphotericin B (24)	4 mg/kg/day intravenously for 5 consecutive days and once per week thereafter for 5 more weeks (total, 10 doses 40 mg/kg).	Prospective, non-comparative, single center
Ritmeijer, 2006, Ethiopia	Miltefosine (63)	100 mg per day for 28 days, orally	Prospective, randomized, open label, multicenter
	Sodium stibogluconate (44)	20 mg/kg per day by intramuscular for 30 days.	
Laguna, 2003, Spain	Amphotericin B lipid complex (17)	total dose of 15 mg/kg , intravenously, 3 mg/kg/day for 5 days	Prospective, randomized, open label
	Amphotericin B lipid complex (20)	total dose of 30 mg/kg , intravenously, 3 mg/kg/day for 10 days	
	Meglumine antimoniate (19)	20 mg Sbv/kg/day, intramuscularly, for 28 days	
Ritmeijer 2001, Ethiopia	Generic sodium stibogluconate or Pentontan (27)	both drugs were given at 20 mg/kg intramuscularly for 30 days	Prospective, randomized, open label
Pintado, 2001, Spain§ #,	Meglumine antimoniate (51)	20 mg Sbv/kg/day (with a maximum daily dose of 850 mg), intramuscularly for 3–4 weeks	Retrospective, historic cohort, single center
Laguna, 1999, Spain	Meglumine antimoniate (44)	20 mg Sbv/kg/day (without an upper dose limit), intramuscularly, for 28 days.	Prospective, randomized, open label, multicenter
	Amphotericin B deoxycholate (45)	0.7 mg/kg/day for 28 days, intravenously	
Behre, 1999, Ethiopia	Meglumine antimoniate (23)	20 mg Sbv/kg/day intramuscularly for 28 days	Prospective, cohort, non-comparative
Delgado, 1999, Spain#	Meglumine antimoniate (25)	20 mg Sbv/kg/day given in 2 separate intramuscular injections (without an upper dose limit)	Retrospective, historic cohort, comparative
Lopéz-Veléz, 1998, Spain¶ #	Meglumine antimoniate (51)	20 mg/Sbv/kg/day given intravenously or intramuscularly for 28 days &	Retrospective, historic cohort, comparative
Laguna, 1997, Spain§	Meglumine antimoniate (29)	≥20 mg Sbv/kg/day for at least 28 days, intramuscularly	Retrospective, historic cohort, comparative
	Liposomal Amphotericin B (4)	NA	
Delgado, 1997, Spain#	Meglumine antimoniate (21)	three-week course 20 mg Sbv/kg/day (with a maximum daily dose of 850 mg), intramuscularly	Retrospective, historic cohort, comparative
	Amphotericin B deoxycholate (20)	0,5 mg/Kg/day (total dose 1–1,5 g)	
Russo, 1996, Spain, Portugal and Italy	Liposomal amphotericin B (10)	Total dose of 40 mg/kg, intravenously, 4 mg/kg on days 1, 2, 3, 4, 5, 10, 17, 24, 31, 38.	Prospective, non-comparative, multicenter
Ribera, 1996, Spain	Meglumine antimoniate (52)	three-week course of daily dose 850 mg, intravenously or intramuscularly	Retrospective, historic cohort non comparative, single center
Rosenthal, 1995, France¶	Meglumine antimoniate (27)	20 mg Sbv/kg/day for at least 21 days, intravenously or intramuscularly	Retrospective, historic cohort comparative, multicenter
	Amphotericin B deoxycholate (14)	≥20 mg/kg total dose, intravenously	
Montalban, 1990, Spain	Meglumine antimoniate (40)	three-week course of daily dose 850 mg, intravenously or intramuscularly	Retrospective, historic cohort non comparative, multicenter

**NA**: information not available **Sbv**: pentavalent antimony.

# in some cases therapy was combined with oral allopurinol (300–1,200 mg/day), or interferon γ (100 µg/m2 subcutaneously) **x˜**: median **μ**: mean **SD**: standard deviation.

¶ one or more treatment arms excluded due to absence separated data.

§ one treatment arm (meglumine antimoniate low dose) excluded: patients data published elsewhere and another (amphotericin B) due to absence separated data.

**Table 2:** Definition criteria used by the studies.

Author, year	Exclusion criteria	VL diagnosis criteria	Clinical cure criteria	Parasitological test of cure
Ritmeijer, 2011	Treatment combination with another antileishmanial drug, patients switched to another treatment due to intolerance	Parasitologically or serologically (rK39 and/or DAT) confirmed, and in few cases clinically defined (negative serological test but strong clinical suspicion of VL and parasitological exam contraindicated).	Fever resolution, spleen regression, hemoglobin increase, and weight gain	Planned for all patients on day 28, it could not be done because of absence of palpable spleen or lymph nodes
Sinha, 2011	NA	Parasitologically or serologically (rK39 and/or DAT) confirmed after exclusion of malaria and bacterial infection.	Clinical improvement	Planned for all patients at 1 month after treatment initiation. It was not performed in clinically cured patients, patients presenting late or material not available
Molina, 2007	Age <18 years, patients without HAART and/or secondary prophylaxis after VL treatment, non-liposomal amphotericin treatment	Parasitologically confirmed	Resolution of fever and improvement of the hematological parameters	Assessment of cure was decided by the attending physician and was performed 1 month after completing treatment
Ritmeijer, 2006	Females, males aged <15 years, severe comorbidity (patients considered to be likely to die during the month's treatment)	Parasitologically or serologically (DAT) confirmed	Clearance of fever, in combination with spleen regression, increased hemoglobin, or weight gain	Splenic or lymph node aspirate performed at day 27–30. In patients without palpable spleen or lymph nodes, cure was established only clinically
Laguna, 2003	Pregnant women, women at risk for pregnancy or were lactating, patients with pancreatitis, prothrombin activity <40%, aminotransferase levels 10× the upper normal limit, myocardiopathy, heart failure, a Qt corrected interval >500 ms, creatinine levels >twice the upper normal limit, allergy to either ABLC or meglumine antimoniate, concomitant treatment with dideoxycytidine or dideoxyinosine and a life expectancy of <6 months.	Parasitologically confirmed	NA	Tissue biopsy sample, taken from the organ used at inclusion (bone marrow, spleen or liver) between 1 and 7 weeks after the completion of therapy
Ritmeijer, 2001	Patients previously treated for VL	Parasitologically or serologically (rK39 and/or DAT) confirmed and in few cases clinically defined (negative serological test but strong clinical suspicion of VL and parasitological exam contraindicated)	Resolution of fever, spleen regression, and weight gain	Performed in patients with splenomegaly at day 25–30
Pintado, 2001	NA	Parasitologically confirmed and in few cases clinically defined (suggestive clinical features, significant serologic titers, and response to specific treatment)	Resolution of fever, improvement of the pancytopenia and hepatosplenomegaly, and absence of symptoms 1 month after the end of treatment	Performed in some patients within first month after the completion of treatment
Laguna, 1999	Age <18 years, pregnant women, history of hepatic encephalopathy, ascites, pancreatitis, myocardiopathy or heart failure, prothrombin time of 20 s or greater, aminotransferase levels 10 times the upper normal limit or higher, or a serum creatinine level above 2 mg/dl.	Parasitologically confirmed	Resolution of fever, improvement of hematological values and regression in the size of the spleen (when it was palpable)	Planned for all patients 4 weeks and 12 months after the completion of therapy
Behre, 1999	Other obvious concurrent infectious diseases	Parasitologically confirmed	Improvement of general condition, reduction in spleen size	Performed in all patients after completion of anti-Leishmania chemotherapy
Delgado, 1999	Non antimonial treatment	Parasitologically confirmed	Resolution of all symptoms and signs attributable to VL	Performed in some patients following completion of therapy
Lopéz-Veléz, 1998	Treatment response not available	Parasitologically confirmed	Resolution of fever, improvement of hematological and hepatosplenomegaly, and absence of symptoms 3 weeks after the end of treatment	Performed (without any selective criteria) on some patients one month after completing the treatment
Laguna, 1997	NA	Parasitologically confirmed	Remission of clinical symptoms potentially due to VL and the absence of any VL relapse for at least six months after treatment.	Criteria used not available
Delgado, 1997	NA	Parasitologically confirmed	Remission of clinical symptoms potentially due to VL	NA
Russo, 1996	NA	Parasitologically confirmed	Improvements in weight, albumin, pancytopenia, and erythrocyte sedimentation rate	Performed on day 45 using criteria not reported
Ribera, 1996	Age <14 years	Parasitologically confirmed	Normalization of all the clinical and hematological parameters	Performed in all patients who showed any persistent clinical or hematological alterations after treatment and in some cases with recovery clinical criteria
Rosenthal, 1995	Incomplete treatment course	Parasitologically confirmed (one case was serologically confirmed by Western Blot)	Disappearance of all clinical signs potentially due to VL	Not reported in some patients because of absence of clinical or microbiological data
Montalban, 1990	NA	Parasitologically confirmed	NA	NA

**NA**: information not available **VL**: visceral leishmaniasis **Parasitologically confirmed:** identification of *Leishmania* amastigotes by direct examination or by isolation of promastigotes in culture of tissue samples **NA**: information not available.

VL was diagnosed in 10 studies exclusively if patients had a compatible clinical illness and positive Giemsa-stained smears or culture for *Leishmania* in samples taken in most cases from the bone marrow, spleen or liver. Few diagnoses were established by the finding of *Leishmania* spp. in biopsy of an unusual site such skin, tongue and gingival mucosae [29] or after staining and/or culture of the buffy coat [20]. Five authors [6], [23], [25], [26], [30] accepted VL diagnosis based on a positive serological result (Western Blot, direct agglutination or rK39 dipstick tests) and in some studies [6], [26] [32], diagnosis was based on clinical grounds alone (negative serological tests, strong clinical suspicion and parasitological test contraindicated).

The efficacy of therapy was assessed by clinical and/or microbiologic criteria. The clinical response definition varied among studies. The remission of fever, improvement of hematological values and regression in the size of the spleen were the main signs observed. A complete resolution of all clinical and hematological parameters [16], [28], [30] or the absence of recurrence in subsequent months after VL treatment [17], [32] was required by some authors to establish clinical cure while all the others defined response as the improvement of the signs and symptoms attributed to disease at the end of treatment, even partial.

A VL episode was considered microbiologically cured when the organ used at inclusion and obtained again usually between days 25 and 45 after initiation of therapy yielded no demonstrable amastigotes by direct visualization or culture. A parasitological control study was planned for all patients in three prospective trials [21], [22], [27]. Only two studies did not report parasitological test of cure [18], [29]. In several centers [6], [23], [25], [26] parasitological test of cure have been routinely performed after treatment, except in those patients without splenomegaly and lymphadenopathy or contraindication to the procedure. In another study [16], the procedure was carried on patients who showed any persistent clinical or hematological alterations after treatment and finally there were those where TOC was performed in some patients after completion of therapy without any disclaimed selective criteria. The percentage of VL episodes tested for parasitological cure ranged from 7.4 to 100% among studies. Overall, 62.3% (456) of the 732 treated episodes with available information about TOC were evaluated with a parasitological test at the end of treatment. Fourteen studies reported number of patients lost to follow up: in 13 (93%) of them it was less than 10%. The follow up length (mean or median reported by fifteen studies) varied between 5 and 14 months (Table 3).

**Table 3:** Main patient characteristics.

Author, year, country	Treatment arms (number of episodes treated)	Age (min-max) years ± SD	Male/female	CD4 cell count (min-max) cells/mm^3^	Antiretroviral therapy use (% of patients)	Previous AIDS diagnosis (% of patients)	% first VL episode	Follow up (interquartile range or standard deviation) in months	Lost to follow up (%)	Treated patients with parasitological test of cure (%)
Ritmeijer, 2011, Ethiopia	Liposomal amphotericin B (195)	μ = 30 (15–56)	179/16	X˜ = 155±123	42/87 (48.3)	NA	59.5	NA	3/195 (1.5)	129/195 (66.1)
Sinha, 2011, India	Liposomal amphotericin B (55)	μ = 35 (30–40)	46/9	μ = 66 (38–112)	0/55 (0)	NA	50.9	x∼ 11.4 (3.4–20.3)	0/55 (0)	43/55 (78.2)
Molina, 2007, Spain	Liposomal amphotericin B (24)	μ = 36 (26–53)	14/1	x˜ = 100 (4–300)	13/15 (86.7)	7/12 (58.3)	57	x∼ 14 (5–44)	2/24 (8.3)	12/24 (50)
Ritmeijer, 2006, Ethiopia	Miltefosine (63)	x˜ = 33,4±9,5	NA	NA	NA	NA	NA	6	NA	NA
	Sodium stibogluconate (44)									
Laguna, 2003, Spain	Amphotericin B lipid complex (17)	x˜ = 34,1±6	15/3	x˜ = 41 (5–366)	5/18 (27.8)	10/18 (55.6)	100	5	5/56 (8.9)	43/56 (76.7)
	Amphotericin B lipid complex (20)	x˜ = 33,7±5,8	17/3	x˜ = 56 (0–731)	13/20 (65)	13/20 (65)				
	Meglumine antimoniate (19)	x˜ = 34±5,2	16/3	x˜ = 26 (1–600)	7/19 (36.8)	16/19 (84.2)				
Ritmeijer, 2001, Ethiopia	Generic sodium stibogluconate or Pentontan (27)	x˜ = 29,9	25/2	NA	NA	NA	100	6	1/27 (3.7)	2/27 (7.4)
Pintado, 2001, Spain	Meglumine antimoniate (51)	x˜ = 33,2±8,2	64/16	32/80<200	19/80 (23.8)	43/80 (53.8)	100	μ 13.8 (3–44)	NA	30/51 (58.8)
Laguna, 1999, Spain	Meglumine antimoniate (44)	μ = 31 (19–64)	40/4	μ = 29 (1–203)	NA	28/44 (63.6)	100	X∼ 10.8	6/89 (6.7)	58/89 (65.2)
	Amphotericin B deoxycholate (45)	μ = 32 (22–57)	36/9	μ = 18 (0–231)		28/45 (62.2)				
Behre, 1999, Ethiopia	Meglumine antimoniate (23)	x˜ = 28,6 (20–47)	22/1	NA	0/23 (0)	NA	NA	NA	0/23 (0)	23/23 (100)
Delgado, 1999, Spain	Meglumine antimoniate (25)	x˜ = 30,2 (19–39)	20/4	x˜ = 66,2 (2–307)	0/25 (0)	17/24 (70.8)	96	13.5 (0.25–26)	1/25 (4)	9/25 (36)
Lopéz-Veléz, 1998, Spain	Meglumine antimoniate (51)	x˜ = 31±7,5	46/8	33/54 <100	NA	25/54 (46.3)	NA	6	0/51 (0)	14/51 (27.4)
Laguna, 1997, Spain	Meglumine antimoniate (29)	NA	42/1	34/43 <100	0/33	29/43 (67.4)	70	μ = 10.4 (2–25)	3/33(9.1)	23/33 (69.7)
	Liposomal Amphotericin B (4)									
Delgado, 1997, Spain	Meglumine antimoniate (21)	x˜ = 34,3±5,1	30/1	x˜ = 37,9 (0–130)	NA	18/31 (58)	75.6	μ = 10.9±8.5	NA	0
	Amphotericin B deoxycholate (20)									
Russo, 1996, Spain, Portugal and Italy	Liposomal amphotericin B (10)	x˜ = 31,3 (25–43)	9/1	NA	NA	5/10 (50)	40	μ = 12	0/10 (0)	8/10 (80)
Ribera, 1996, Spain	Meglumine antimoniate (52)	x˜ = 28 (20–64)	NA	x˜ = 26,7 (2–268)	37/52 (71.1)	18/52 (34.6)	71.7	x˜ = 7.5	0/52 (0)	26/52 (50)
Rosenthal, 1995, France	Meglumine antimoniate (27)	x˜ = 34 (22–68)	42/8	μ = 25 (0–200)	NA	21/50 (42)	NA	NA	5/41 (12.2)	36/41 (87.8)
	Amphotericin B deoxycholate (14)									
Montalban, 1990, Spain	Meglumine antimoniate (40)	x˜ = 29,4 (20–53)	36/4	x˜ = 204,3±27,5	NA	19/40 (47.5)	NA	μ = 12,8 (0.5–71)	0/40 (0)	0

**VL:** visceral leishmaniasis **NA**: information not available **x˜**: median **μ**: mean **SD**: standard deviation.

Table 3 shows also the baseline characteristics of included patients. The mean or median age varied between 28 and 36 years among the studies, most patients were male (87.4% of the 748 patients with gender information available) and 49.6% of the patients (269/542) had AIDS criteria before VL diagnosis. Antiretroviral use was reported in 10 studies and in four of them, no patient was on HIV treatment at VL diagnosis. The median or mean baseline CD4 cell counts range from 25 to 204 cells/ml.

Five treatment regimens were reported in 17 studies included in this review. There were 13 studies with 457 VL episodes evaluating antimony compounds: five studies assessing “low antimonial dose” and eight evaluating “high antimonial dose” schedules. Therefore, we assume that a low pentavalent antimony dose was administered when the length of treatment was shorter than 28 days and/or less than 20 mg Sbv/kg/day was administered. High antimonial dose was defined as treatment with ≥20 mg Sbv/Kg/day for at least 28 days. In some studies, the antimonial treatment was combined with allopurinol, or recombinant human interferon-gamma in few patients [20], [28], [29], [31]. Three studies evaluated the response to amphotericin B deoxycholate and 6 studies described the response to one of the lipid formulations of amphotericin B (L-Lip-AmB), which includes liposomal and lipid complex amphotericin, most of them (5 of 6) by using total doses above 25 mg per kg. Only one study evaluated the response to treatment with miltefosine [23].

The Newcastle-Ottawa Scale (NOS) for Assessing the Quality of Nonrandomized Studies is show in Table 4 and the scores ranged from 5–7. As shown in Table 2, there was adequate selection of patients in included studies, as almost all were parasitological confirmed cases who were largely representative of source populations. In ten studies, however, patients were reviewed retrospectively for inclusion, with some risk of bias in the case selection. Seven studies were non comparative and reported only one arm treatment outcome.

**Table 4:** The Newcastle-Ottawa Scale (NOS) for assessing the quality of nonrandomized studies.

Non RTC studies	Selection			Comparability			Assessment of Outcome			Total Quality score
Author, year	Representativeness of treated arm	Selection of the comparative treatment arm (s)	Ascertainment of the treatment regimen	Demonstration that outcome of interest was not present at star of study	Comparability between patients in different treatment arms – main factor: CD4 lymphocytic count	Comparability between patients in different treatment arms – secondary factor: co-morbidities	Assessment of outcome with independency	Adequacy of Follow up length (to assess outcome)	Lost to follow up acceptable (less than 10% and reported)	
Ritmeijer, 2011	*		*	*			*	*	*	6
Sinha, 2011	*		*	*			*	*	*	6
Molina, 2007	*		*	*			*	*	*	6
Pintado, 2001	*		*	*			*	*		5
Laguna, 1999	*		*	*			*	*	*	6
Behre, 1999	*		*	*			*	*	*	6
Delgado, 1999	*		*	*			*	*	*	6
Lopéz-Veléz, 1998	*	*	*	*			*	*	*	7
Laguna, 1997	*	*	*	*			*		*	6
Delgado, 1997	*	*	*	*			*	*		6
Russo, 1996	*		*	*			*	*	*	6
Ribera, 1996	*		*	*			*	*	*	6
Rosenthal, 1995	*	*	*	*			*	*		6
Montalban, 1990	*		*	*			*	*	*	6

**RTC**: randomized controlled trial.

The summarized measures for initial clinical improvement, global cure and death, according to the intention-to-treat analysis, are shown in Table 5. Relapse was assessed including treated patients who were considered cured. To assess parasitological cure rate only patients who underwent test of cure were included.

**Table 5:** Summarized measurements (95% confidence interval) for main outcomes.

Treatment group	Clinical cure rate %	Parasitological cure rate %	Global cure rate %	Early death rate %	Relapse rate %	Serious adverse event rate %	Treatment interruption due to intolerance %
Antimony low dose	58.4 (33.0–80.0)	98.1 (76.4–99.9)	52.2 (30.2–73.3)	7.2 (3.6–14.1)	40.6 (17.2–69.3)	4.8 (0.7–27.1)	10.8 (3.8–27.4)
	I^2^ = 73.8		I^2^ = 0	I^2^ = 0	I^2^ = 0		I^2^ = 36.9
	n = 75 (3 studies)	n = 26 (1 study)	n = 99 (3 studies)	n = 113 (3 studies)	n = 50 (2 studies)	n = 21 (1 study)	n = 124 (3 studies)
Antimony high dose	58.7 (35.9–78.4)	72.6 (55.4–85)	56.1 (40.7–70.4)	18.4 (13.3–25)	25.6 (12.9–44.6)	23.3 (17.4–30.4)	21 (9.2–41.2)
	I^2^ = 87.8	I^2^ = 28.4	I^2^ = 76.9	I^2^ = 44.8	I^2^ = 55.6	I^2^ = 66.3	I^2^ = 80.4
	n = 126 (4 studies)	n = 121 (7 studies)	n = 211 (7 studies)	n = 192 (6 studies)	n = 58 (5 studies)	n = 456 (5 studies)	n = 131 (4 studies)
Amphotericin B deoxycholate	85.0 (41.7–97.8)	62.2 (47.4–75.1)	76.8 (54–90.3)	11.1 (4.7–24.1)	33 (17.6–53.9)	16.6 (9.5–27.2)	10.6 (3.0–31.5)
			I^2^ = 58.5			I^2^ = 0	I^2^ = 0
	n = 20 (1 study)	n = 45 (1 study)	n = 79 (3 studies)	n = 45 (1 study)	n = 24 (1 study)	n = 290 (2 studies)	n = 65 (2 studies)
L-Lip-amB doses <25 mg/Kg	-	37.5 (17.9–62.3)	35.3 (16.8–59.6)	2.8 (0.2–32.2)	50 (12.3–87.7)	2.8 (0.2–32.2)	5.6 (0.8–30.7)
		n = 16 (1 study)	n = 17 (1 study)	n = 17 (1 study)	n = 4 (1 study)	n = 17 (1 study)	n = 18 (1 study)
L-Lip-amB doses >25 mg/Kg	91.6 (74.7–97.6)	72.3 (51.0–86.7)	72.7(56–84.8)	6.1 (3.9–9.4)	39.4 (18.9–64.5)	9.5 (3.5–23.3)	4.2 (1.1–14.9)
	I^2^ = 0	I^2^ = 74.6	I^2^ = 80.7	I^2^ = 0	I^2^ = 74.2	I^2^ = 0	I^2^ = 0
	n = 89 (3 studies)	n = 213 (6 studies)	n = 318 (6 studies)	n = 308 (6 studies)	n = 68 (4 studies)	n = 51 (3 studies)	n = 106 (4 studies)
Miltefosine	-	-	77.8 (65.9–86.4)	1.6 (0.2–10.4)	28.6 (18.3–41.7)	24.6 (17.9–32.9)	0.8 (0.0–11.3)
			n = 63 (1 study)	n = 49 (1 study)	n = 56 (1 study)	n = 126 (1 study)	n = 63 (1 study)

I^2^ values of <25%, 25 to 50% and >50% indicate mild, moderate and substantial heterogeneity, respectively n: number of patients available L-Lip-amB: Lipid formulations of amphotericin B.

**Figure 2:**
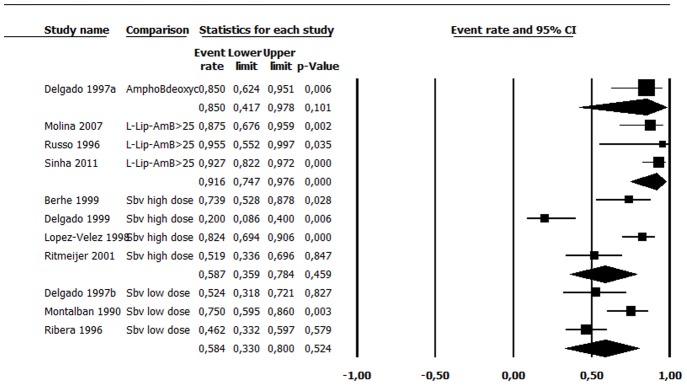
Clinical improvement rate. Egger's test for publication bias (all studies): p = 0.18 Statistical heterogeneity: I^2^ (L-Lip-AmB>25) = 0; I^2^ (Sbv high dose) = 87.8; I^2^ (Sbv low dose) = 73.

Clinical improvement rate using amphotericin in lipid formulation (L-Lip-AmB) was superior compared to both antimony therapy groups (Figure 2).The unique study herein included using deoxycholate amphotericin B also exhibited a clinical response rate (85%, 95%CI 41.7–97.8%) similar to L-Lip-AmB group (91.6%, 95%CI 74.7–97.6%). Therefore, it was not possible to attest the presence of a difference in performance among several amphotericin B formulations. The global and parasitological cure rates varied widely within the same treatment arm, which hampered any indirect comparison between them (Table 5). This fact probably reflects different criteria used by the studies to perform test of cure. However, it is worth mentioning that the difference by indirect comparison from 76% to 56% (wide confidence intervals) in global cure rate between deoxycholate amphotericin B and Sbv treatment groups, respectively, although heterogeneous, was confirmed by direct comparison of the studies [29], [30] which actually compared these two treatment arms (OR 6,08 for amphotericin superiority 95%CI 1,99–18,5; I^2^ 0%).

Regarding tolerance, the difference in adverse event rate between high dose of Sb^v^ (23.3; 95%CI 17.4–30.4) and lipid formulation of amphotericin B (9.5; 95%CI 3.5–23.3) seems to be relevant, despite the overlap between the confidence intervals observed (Table 5). In agreement with this, the rate of early discontinuation of therapy due to toxicity also seems to be higher with Sb^v^ than with lipid formulation of amphotericin B. All these outcomes were adjusted for CD4 lymphocyte count, which had no influence on treatment effect as evaluated by meta-regression.

**Figure 3:**
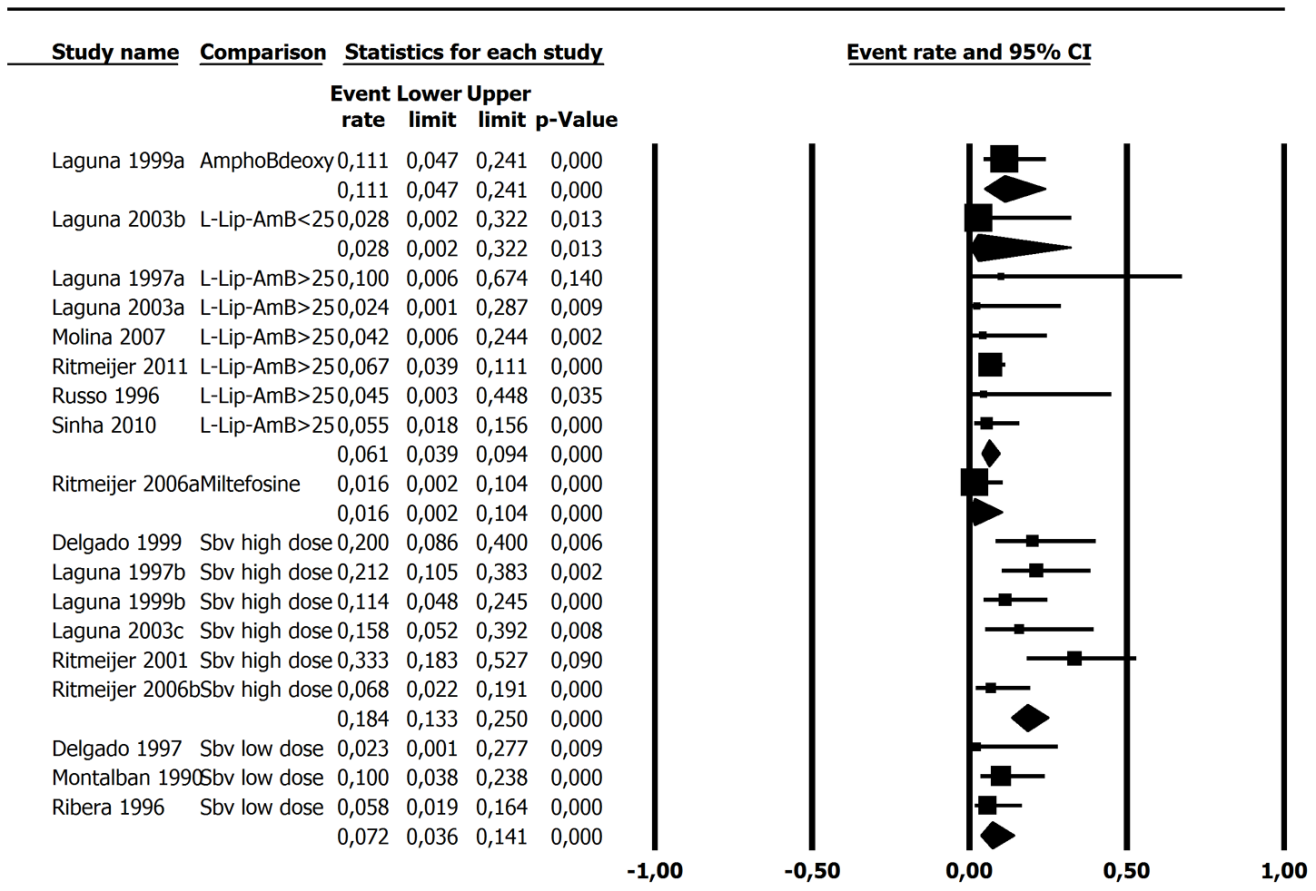
Death rate. Egger's test for publication bias (all studies): p = 0.20 Statistical heterogeneity: I^2^ (L-Lip-AmB>25) = 0; I^2^ (Sbv high dose) = 44.8; I^2^ (Sbv low dose) = 0.

**Figure 4:**
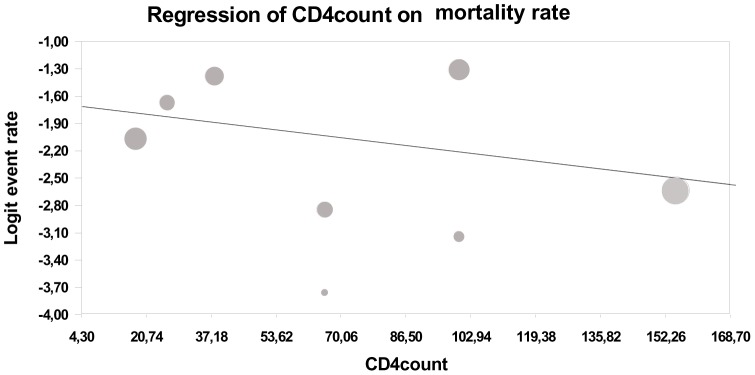
Meta-regression between death rate and CD4 lymphocytes count. p = 0.18.

The most outstanding difference in outcome between the treatment regimens was observed in early mortality rate: about 3 times higher in high-dose antimony (18.4.%, 95%CI 13.3–25%) in comparison to L-Lip-AmB (6.1%, 95%CI 3.9–9.4%) treated patients, without overlap of confidence intervals (Table 5 and Figure 3). Meta-regression revealed no influence of CD4 lymphocyte count in death rate related to different treatments (Figure 4).

A variety of adverse effects were depicted, such as vomiting, diarrhea, anemia, eletrolic disturbs, pancreatic, cardiac, hepatic and renal dysfunction (Table 6). The events reported were not sufficiently similar to allow a meta-analysis of adverse effects. In two studies [22] [27] adverse reactions were scored according to the World Health Organization (WHO) scale for toxicity. Six studies did not report on the occurrence of reactions to VL therapy and one study [17] reported the occurrence of adverse events without discriminating the type of treatment received.

**Table 6:** Adverse events reported.

Author, year	Adverse events
Ritmeijer, 2011	NA
Sinha, 2011	NA
Molina, 2007	Nonsystematic description of the adverse effects observed. Only impairment of renal function was reported.
Ritmeijer, 2006	It were reported: bleeding, diarrhea, vomiting, pneumonia, death, default#
Laguna, 2003	Adverse reactions were scored according to the World Health Organization (WHO) scale for toxicity. Adverse events were considered to be toxicity of grade 2 or greater
Ritmeijer, 2001	It were reported: bleeding, diarrhea, vomiting, pneumonia, death
Pintado, 2001	NA
Laguna, 1999	Adverse reactions were scored according to the World Health Organization (WHO) scale for toxicity. Adverse events were considered to be toxicity of grade 2 or greater
Behre, 1999	NA
Delgado, 1999	It were reported: hyperamylasemia, acute pancreatitis, serum creatinine >2 mg/dl, leukocyte count <1,500 cells/ml, T wave inversion, vomiting
Lopéz-Veléz, 1998	It were reported the following serious adverse effects: anemia (defined as a 25% reduction in the hematocrit), renal toxicity (a three-fold increase in the normal level of serum creatinine), hepatic toxicity (a ten-fold increase in the base values of the transaminases), and hyperamylasemia (a two-fold increase in normal serum amylase values)
Laguna, 1997	NA
Delgado, 1997	Nonsystematic description of the adverse effects observed
Russo, 1996	Nonsystematic description of the adverse effects observed
Ribera, 1996	NA
Rosenthal, 1995	NA
Montalban, 1990	NA

NA: information not available.

# defined as starting but failing to complete treatment because of reasons other than death or decision by the clinician.

Ten out seventeen studies reported the VL relapse rate without secondary prophylaxis and it ranged from 26 to 50%. VL relapse was diagnosed if parasites were observed in tissue samples after initial clinical cure. It was not possible to attest the presence of any difference in relapse among different treatments.

## Discussion

Available evidence suggests superiority of amphotericin B in the treatment of HIV-infected patients with visceral leishmaniasis (VL-HIV). The main conclusion of this review is the higher mortality rate among VL-HIV patients treated with Sb^v^ than among patients treated with amphotericin B. It could be due to the low efficacy or the toxic effects of antimony; however the risk of death seems to be related to the increase in Sb^v^ dose, suggesting that toxicity is the most important factor. Our data confirm that antimony compounds are poorly tolerated in the presence of HIV infection, as clearly demonstrated by the study evaluating mortality among coinfected and HIV-uninfected, both treated with Sb^v^ [23]. The higher mortality related to Sb^v^ than that observed with miltefosine in HIV-infected patients strongly suggests that this effect was caused by the antimonial treatment itself. Pentavalent antimonial drugs have been used for the treatment of VL since the 1940s [33]. Sodium stibogluconate (brand name Pentostam [GSK]); also generic versions from many manufacturers) and meglumine antimoniate (brand name Glucantime [Aventis]) remain the most widely used antileishmanial agents [33]. The mechanism of action of pentavalent antimonial drugs is uncertain; *in vivo* conversion to trivalent antimony compounds may be involved in both antileishmanial activity and drug toxicity [34]. Other studies suggest that antileishmanial activity may occur via inhibition of parasite ADP phosphorylation, DNA I topoisomerase, and/or trypanothione reductase [35]. The compounds are well known for their toxicities such as severe vomiting, arrhythmia and pancreatitis, besides emerging drug resistant. Doses below those currently used (such as three weeks course with a maximum daily dose of 850 mg of Sb^v^ in adults) have been used in the past [18], [30], [31] but were abandoned by lower efficacy compared to 20 mg/kg/day of Sb^v^ for a minimum of 28 days, which was also suggested by our data. In this review, it was also observed that, as is already accepted [36], [37], toxicity is directly related to the increase in the dose of Sb^v^, verified by an increase in the occurrence of severe events and mortality rate. Although sensitivity to specific drugs varies by region, it is unlikely that these regional differences and strains of *Leishmania* have contributed to the discrepancy observed in clinical response since all except one study were performed in Europe and Ethiopia, where resistance is rare. Only one study performed in India was included and, in this case, the treatment was carried out with liposomal amphotericin B and it reached a clinical response rate of 93% [25]. Meanwhile it is not possible to certify that other variables related to the characteristics of patients in different countries, such as comorbidities or degree of immunosuppression, or to clinical spectrum of the disease, have influenced the efficacy and mortality results.

All but one study in the L-Lip-AmB group used liposomal formulation as treatment. Liposomal amphotericin B (AmBisome, Gilead) consists of amphotericin B packaged with cholesterol and other phospholipids within a small unilamellar liposome. The liposomal drug formulation has improved stability in blood, macrophages, and tissues, permitting more effective tissue penetration with sustained tissue drug levels, especially in the liver and spleen. This formulation has increased affinity for ergosterol and its precursors. In addition, the presence of cholesterol in the formulation minimizes interaction with mammalian cell membranes, thereby reducing toxicity [38]. Because cost is the limiting factor for use of liposomal amphotericin B, many different regimens have been evaluated in an attempt to find the lowest total dose with acceptable efficacy. Due to the small number of studies in this review, it was not possible to compare schemes with different doses or preparations of amphotericin B. Our data suggest, however, that liposomal and lipid complex preparations are better tolerated than conventional amphotericin B or pentavalent antimony, and this probably contributes to their highest rate of clinical efficacy. Current research is now shifting away from developing optimized regimens of existing drugs toward demonstrating their implementation is feasible in the field [39]. There is a clear trend among experts that antileishmanial therapy in endemic regions should move toward combination drug regimens based on the following rationale: (1) protect the limited armamentarium of antileishmanial agents from development of acquired resistance and (2) establish shorter treatment courses with high efficacy to improve compliance and decrease treatment costs [36]. Drugs for Neglected Diseases initiative (DNDi), a collaborative, patients' needs-driven, non-profit drug research and development organization is currently funding several studies in Eastern Africa, including two randomized, open-label clinical trials assessing the safety and efficacy of combination regimens: SSG plus single dose AmBisome, miltefosine plus single dose AmBisome (ClinicalTrials.gov NCT01067443) and SSG plus paramomycin sulphate (ClinicalTrials.gov NCT00255567). As far as we know, there is no registered trial evaluating combination therapy for *Leishmania*-HIV coinfected patients.

### Limitations

The main limitation of this review is the paucity of quality evidence. On the other hand, four literature databases were searched, making it a comprehensive review. Clinical decisions must be made. To aid in this task we presented indirect comparisons, including non-randomized studies, in the same way others have done [40], as a tool to synthesize the available information. By adjusting indirect comparison for CD4 lymphocyte count, we have evaluated an important confounder factor for mortality rate. To our knowledge, no systematic review has investigated the comparative efficacy of the several treatment options for VL-HIV patients.

When there is no or insufficient direct evidence from randomized trials, the adjusted indirect comparison may provide useful or supplementary information on the relative efficacy of competing interventions. The validity of the adjusted indirect comparisons depends on the internal validity and similarity of the included trials [41]. Ideally, direct and indirect estimates should be combined in mixed treatment comparisons only after adequate assessment of the consistency of the evidence [42]. In this case, evidence of consistency is the correlation observed between indirect comparison performed (comparison among patients treated with different schemes in different studies) and the only direct comparison that could be made (two studies comparing the same two treatment arms). Other important qualitative features include the degree of similarity of populations, interventions, outcomes, study objectives and study designs that incorporate both clinical and biological plausibility.

Many studies used selective criteria excluding patients with more severe clinical conditions or with high risk of toxicity, such as those with renal, pancreatic or heart dysfunction. This methodological choice could have influenced toward a lower rate of adverse events and a higher percentage of therapeutic success. However, the more stringent studies were also comparative and randomized studies [22], [23], [27], so, such selection affected equally all treatment arms. Similarly, studies with highly demanding criteria of cure, as those requiring complete resolution of symptoms [16], [30] may have had the therapeutic success rates underestimated.

In all studies, the outcome ascertainment was not blinded. Indeed, either the participants or the researchers who collected disclosure information may have been aware of participants' disease status at the time of data extraction. However, most studies have clearly defined criteria for establishing cure, and in many of them parasite clearance was required. The time to cure assessment and the follow-up time was relatively uniform and adequate in all studies. So we performed the summary measures of effectiveness for each treatment regimen to perform an indirect efficacy comparison. To carry out a clinically sound analysis, we used a conservative approach and imputed outcomes for the missing and discontinued participants assuming that they did not respond to treatment. Therefore, no response includes the intrinsic lack of efficacy and toxicity limiting the completeness of the treatment. In fact, in this review there was an inverse association between adverse events rate and clinical response, as expected. Although parasitological cure rate could theoretically provide reliable information about treatment efficacy, in most studies post treatment TOC was performed only in patients with uncertain clinical response, which represents a selection bias that could underestimate response rates.

In conclusion, these indirect comparisons suggest higher clinical response rate with amphotericin B than with antimony treatment, which appears to be related to less toxicity than with higher effectiveness of lipid formulations of amphotericin. Antimonial therapy carries a higher rate of drug discontinuation and a significantly higher mortality indirectly compared to treatment with amphotericin B. A relatively large body of non-comparative cohort studies supports, at this time, the use of amphotericin B as the first choice for VL treatment in HIV-infected patients. The optimal dose of amphotericin and the difference in efficacy between its various formulations remain to be established.

## Supporting Information

Checklist S1 PRISMA Checklist.
(DOC)
